# Vitiligo-Like Depigmentation Induced by Cyclin-Dependent Kinase 4/6 Inhibitors in the Treatment of Metastatic Breast Cancer: A Case Report and Literature Review

**DOI:** 10.7759/cureus.83513

**Published:** 2025-05-05

**Authors:** Chuan Yaw Lee, Lydia Tang-Lin, Sok Yuen Beh

**Affiliations:** 1 Division of Medical Oncology, National Cancer Centre Singapore, Singapore, SGP; 2 Department of Dermatology, Changi General Hospital, Singapore, SGP

**Keywords:** abemaciclib, breast cancer, cdk 4/6 inhibitors, cutaneous toxicities, palbociclib, ribocilib, vitiligo, vitiligo-like depigmentation, vitiligo-like lesions

## Abstract

Cyclin-dependent kinase (CDK) 4/6 inhibitors are commonly used in the first-line treatment of hormone receptor-positive, human epidermal growth factor receptor 2-negative metastatic breast cancer, and more recently in the adjuvant setting as well. Whilst cutaneous toxicities are well documented, vitiligo-like depigmentation (VLD) is rarely reported in existing literature. Such adverse effects impair tolerability and compromise patients’ quality of life. Here we describe a case from our centre of a 72-year-old Chinese woman with recurrent, metastatic hormone-positive breast cancer who developed a rare case of VLD after eight months of treatment with ribociclib. The development of lesions was preceded by diffuse pruritis, initially beginning on her back before eventually involving her distal limbs. Ribociclib was switched to palbociclib, which was subsequently stopped as the VLD continued to progress. She was reviewed by dermatology, with CDK 4/6 inhibitor-induced VLD as a clinical diagnosis. She was started on topical tacrolimus and mometasone cream with minimal improvement. Our case, in conjunction with the 40 cases evaluated as part of our literature review, highlights the need to increase physician awareness of VLD as a rare but significant complication of CDK 4/6 inhibitor use. Given its expanding role in everyday practice, it would be useful to recognise its typical clinical course and morphology, understand its pathophysiology and explore potential treatment options.

## Introduction

In the ever-evolving landscape of breast cancer treatment, cyclin-dependent kinase (CDK) inhibitors have become entrenched in the management of hormone receptor-positive, human epidermal growth factor receptor 2-negative (HER2-) tumours. These novel targeted therapeutic agents inhibit cyclin-D/CDK4/6 complex activity and result in hyperphosphorylation of the retinoblastoma protein. This remains bound to the early region 2 binding factor (E2F) transcription factor and blocks cell cycle progression from the G1 to the S phase.

Palbociclib, ribociclib and abemaciclib have received international regulatory approval for use in combination with endocrine therapy in the metastatic and recurrent setting. They confer a progression-free and overall survival benefit when added to a hormonal backbone [1]. More recently, the latter two have additionally been approved as an adjuvant strategy [2]. Given its growing role in various treatment paradigms, it is essential physicians are comfortable in managing the related toxicities and are aware of less common adverse effects.

CDK 4/6 inhibitors are usually well tolerated. The most common toxicities include that of myelosuppression, fatigue, nausea and diarrhoea. Cutaneous toxicities occur in 20-30% of patients with only a small proportion being grade 3 or 4. These include mainly alopecia, maculopapular rash and pruritis. These toxicities compromise tolerability and could potentially result in discontinuation of treatment. There have also been more severe cutaneous adverse effects of Stevens-Johnson syndrome, toxic epidermal necrolysis and Sweet syndrome reported in the literature, but these constitute less than 1% [3].

Vitiligo is a chronic acquired, autoimmune pigmentary skin disorder characterised by the absence of pigmentary cells and loss of melanocytes from the epidermis. Its clinical presentation is well-demarcated white patches on the skin with a typically symmetrical distribution, of variable sizes and shapes. Drug-induced vitiligo or vitiligo-like depigmentation (VLD) is a well-described variant. In terms of anti-cancer treatment, immune checkpoint inhibitors and anti-tumour necrosis factor agents are the more commonly reported culprits given vitiligo’s immunologic pathogenesis. Other culprits include tyrosine kinase inhibitors such as cabozantanib and pazopanib [4]. VLD is a rare but recognised adverse effect of CDK 4/6 inhibitors that remains poorly understood to date. Whilst not usually debilitating or overtly symptomatic, it can lead to stigmatisation, mental distress and cosmetic detriment. This impairs patients’ quality of life while on treatment. Here we examine a case of CDK 4/6 inhibitor-induced VLD in our centre and conduct a review of the 40 reported cases in existing literature of vitiligo-like reactions from CDK4/6 inhibitor use in breast cancer.

Methodology

We reviewed various published case reports, case series, and meta-analyses on the use of CDK4/6 inhibitors for reported cutaneous adverse events. The literature review was conducted utilising PubMed utilising the following keywords: CDK4/6 inhibitors, ribociclib, palbociclib, abemaciclib, vitiligo, vitiligo-like lesions, vitiligo-like depigmentation, breast cancer, cutaneous toxicities, skin adverse events. We were able to include nine case reports, two case series, two retrospective reviews, and one case study with a literature review.

## Case presentation

Our case is that of a 72-year-old Chinese woman of good performance status, who presented with a fungating breast mass in February of 2017. She had comorbidities of hypertension, hypercholesterolemia, and pulmonary tuberculosis that was treated in her 20s. There was no known personal or family history of autoimmune disease or dermopathies. 

She was diagnosed with locally advanced, stage III (cT4N0M0) grade 2 invasive ductal carcinoma that was hormone receptor-positive, HER2-. She subsequently underwent neoadjuvant chemotherapy with a third-generation regimen of four cycles of doxorubicin and cyclophosphamide and 12 weeks of paclitaxel. She had a good clinical and pathological response and went for a simple mastectomy with axillary clearance. She was adherent to postoperative follow-up, completed radiotherapy, and was started on adjuvant letrozole. Four years later, in April 2021, she was incidentally noted to have raised tumour markers with scans showing new multifocal hepatic metastases. She was started on first-line capecitabine with a radiological response; however, she tolerated it poorly and was de-escalated to tamoxifen monotherapy after just three months. She progressed eventually after 21 months on tamoxifen alone and was started on fulvestrant and ribociclib 600 mg once daily in March 2023. She tolerated fairly with dose reduction of ribociclib to 400 mg in view of grade 3 neutropenia.

**Figure 1:**
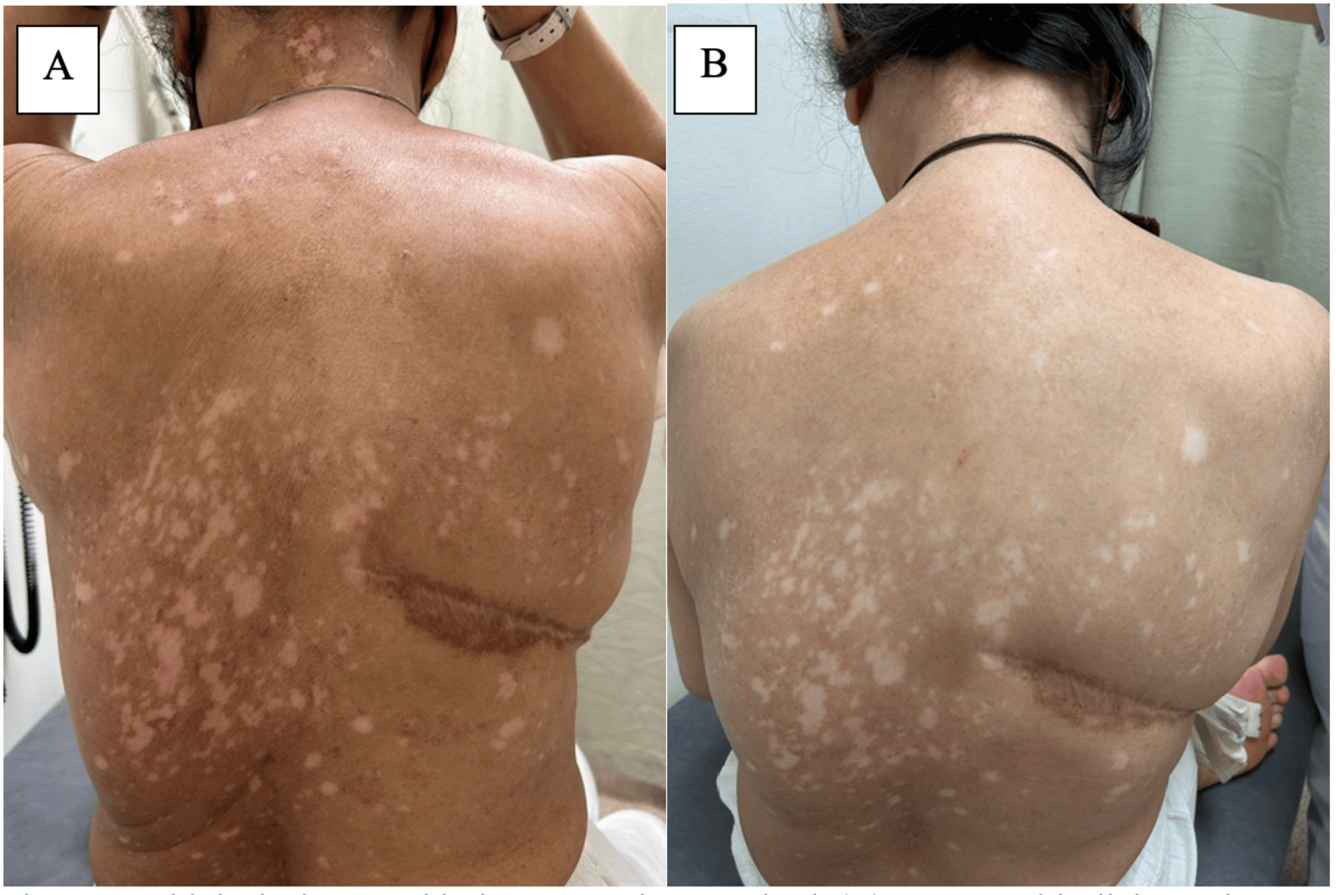
Multiple depigmented lesions on neck, upper back (A) at onset with slight erythema and pruritus (B) after cessation of CDK 4/6 inhibitors and application of topicals CDK: cyclin-dependent kinase

**Figure 2:**
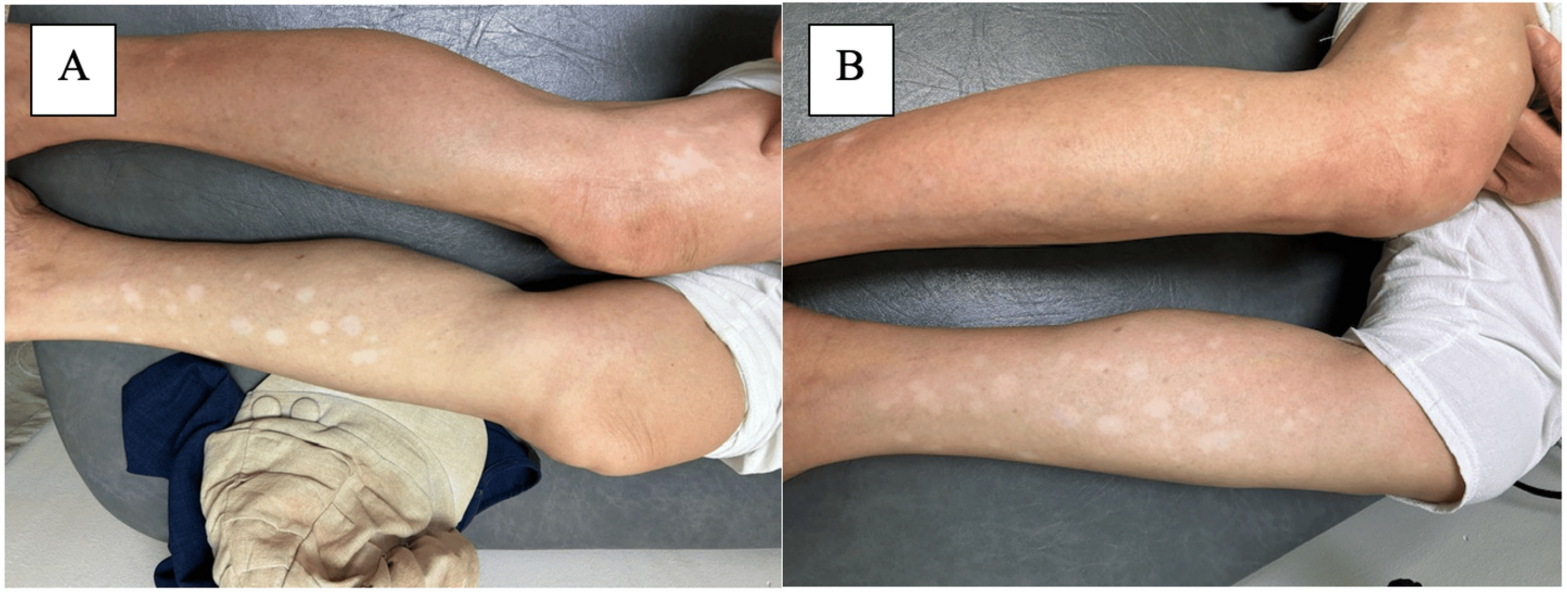
Well demarcated, depigmented patches of varying sizes over anterior shin and lateral thigh (A) at onset (B) after cessation of CDK 4/6 inhibitors and application of topicals CDK: cyclin-dependent kinase

After eight months on ribociclib, she started to develop generalised pruritis with no discernible rash. Scans continued to show sustained response and she was continued on the regimen. In February 2024, after 11 months on ribociclib, she started to develop diffuse hypopigmentation over her entire back (Figure 1A) which eventually involved her distal limbs, lateral thigh and anterior shin (Figure 2A).

**Figure 3:**
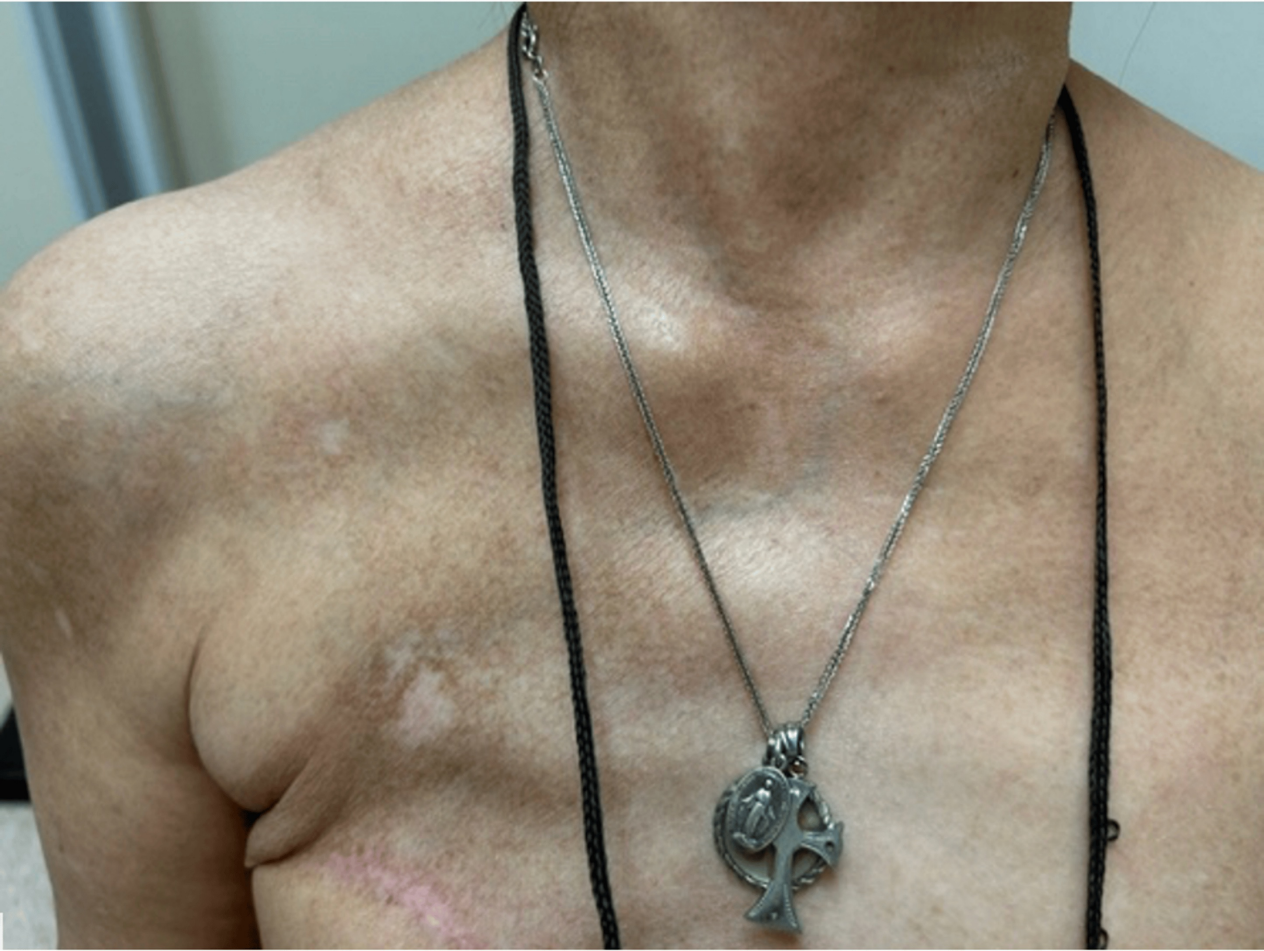
White patches with hyperpigmented edges on anterior chest wall

Some lesions were also noted on her anterior chest wall (Figure 3).

Ribociclib was stopped in April and changed to palbociclib instead, but in view of persistent and progressive lesions, CDK 4/6 inhibitors were ceased entirely in May 2024 and she was continued on fulvestrant alone. Her vitiligo-like lesions have stayed stable since (Figure 1B, 2B).

She was referred to dermatology and the impression was CDK 4/6 inhibitor-induced VLD. Other differentials considered included post-traumatic or chemical-induced leukoderma, para-malignant hypomelanoses and post-inflammatory hypopigmentation. She was offered a skin biopsy to confirm the diagnosis but was not keen. She was started on a trial of topical tacrolimus and mometasone cream with minimal improvement. In October 2024, her scans showed progressive disease after a progression-free interval of 19 months, and after a discussion with the patient, she has since been restarted on ribociclib. As of March 2025, her lesions have remained stable albeit non-resolving.

## Discussion

Our patient adds to the existing 40 cases of VLD described in various case reports and case series since the incorporation of CDK 4/6 inhibitors into our treatment algorithm for hormone receptor-positive, HER2-negative breast cancer. The exact incidence of VLD in patients on CDK 4/6 inhibitors is not known. VLD was seen in up to 3% of patients on ribociclib in the MONALEESA-7 trial [5], with a retrospective analysis by Sollena et al. stating only 0.007% in their patient group [6]. There seem to be no patient demographic or clinical characteristics that show a preponderance to the development of such lesions. Patients ranged from 37 to 86 years of age with a median age of 58.5 years. Of the 41 cases, only two were reported to have a personal history of autoimmune comorbidities (one alopecia areata, one pre-existing non-segmental vitiligo). None had a family history of such autoimmune conditions. Of the available information on the ethnicity of 12 patients, nine were Caucasian and three were Asian, one of whom was reported as of Indian heritage.

Based on the available literature appraised, the majority of cases described are in patients on ribociclib (29 of 41, 70.7%), with fewer cases being reported on palbociclib (nine of 41, 21.9%) and abemaciclib (three of 41, 7.3%). The higher reported VLD rates with ribociclib in this case series mirrors that of the US FDA adverse event reporting system (FAERS) post-marketing monitoring. Of interest, the recently developed dalpiciclib out of China, used in the treatment of melanoma, has also described skin hypopigmentation as a reported toxicity, suggesting a potential class effect [7].

Letrozole was the most common hormonal treatment partner employed (30 of 41, 73.2%), with others being fulvestrant, anastrozole and exemestane. While these anti-estrogen agents may cause cutaneous adverse events, resultant VLD has not been reported in existing literature. There is not enough data to suggest if any particular hormonal partner would increase the risk of VLD development. All patients were being treated for metastatic disease as the available literature predates the approval of adjuvant abemaciclib and ribociclib.

As per our case, the majority of patients seem to develop pruritis as a preceding symptom (23 of 41, 56.1%), while two had an erythematous rash of which one was vesicular. Of note, this is significantly higher than the incidence of pruritis in all vitiligo patients, reported to be just 20%. The onset of vitiligo ranged from as early as one month on treatment to 28 months after commencement, with the median time to onset being eight months. Variable time of onset reported could be contributed to by delayed diagnosis or under-reporting of symptoms given the rarity of this adverse effect. 

The most common distribution of the lesions was involving the limbs and extremities (34 of 41, 82.9%), face (19 of 41, 46.3%), followed by chest (15 of 41, 36.6%) and back (11 of 41, 26.8%). These suggest a predilection to sun-exposed areas initially, followed by a spread to less commonly affected areas, congruent with existing literature. In one reported case, it was further associated with photo-allergic dermatitis [8]. Typically, the lesions presented clinically as depigmented macules and patches of irregular sizes and demarcation. Wood lamp examination showed blue-white patches with clear boundaries and increased pigmentation at the edges of the patches. Cases were diagnosed mainly on clinical examination.

Vitiligo remains a clinical diagnosis with biopsy not being required, but sometimes helpful to rule out differential diagnosis. Of the 41 reported cases, only six underwent a skin biopsy. Findings included perivascular inflammatory changes and significant loss of melanocytes which are congruent with the usual findings from skin biopsy of vitiligo patients, but also mild lymphocytic infiltrate along the basement membrane layer and focal melanin pigment incontinence that are not classical vitiligo features. These are instead seen in interface dermatitis, which can be associated with drug-induced lichenoid reactions, connective tissue diseases and polymorphous light eruptions. This may be supportive of the theorised pathogenesis of VLD from CDK4/6 inhibitors where there is a loss of immune tolerance leading to an active inflammatory response leading to melanocyte destruction and apoptosis.

There are multiple proposed pathogenesis of VLD resulting from CDK4/6 inhibition in itself. CDK inhibition in keratinocytes causes small ubiquitin-like modifier (SUMO) dysregulation and resultant loss of keratinocyte survival stimuli to the melanocytes which leads to destruction [9]. CDK inhibition can also result in a pro-apoptotic effect and increased melanocyte senescence [10]. Additionally, CDK 4/6 inhibitors promote the cytotoxic activity of effector T-cells via the nuclear factor of activated T cells (NFAT) upregulation and also by inhibition of regulatory T-cells [11]. Ultraviolet B (UVB) radiation induces DNA damage already compounded by CDK 4/6 inhibition, explaining the distribution of such lesions in sun-exposed regions [12,13].

The relationship between prognosis and the appearance of such lesions is unknown. The occurrence of vitiligo in melanoma and renal cell carcinoma patients on immunotherapy has been associated with favourable outcomes [3]. Additionally, existing literature describes an association between the incidence of treatment-related toxicities and better clinical outcomes of certain cancer drugs including immunotherapy. It is unclear if this is similarly reflected in CDK 4/6 inhibitors and our examined patient population. Of the available cases, progression-free survival (PFS) duration was reported in 16 patients with seven still undergoing treatment with CDK 4/6 inhibitors. Ten of 16 (62.5%) had a PFS of > 24 months, higher than the PFS reported in the first-line metastatic setting studies. A case series by Pasqualoni et al. also reported two cases of patients who developed VLD, both with a long PFS of 39 and 50 months [14]. These findings should be further explored in future studies and may increase the impetus of physicians to treat through VLD should it occur.

There are no standard treatment guidelines available in the treatment of VLD induced by CDK 4/6 inhibitors. Anti-histamines can be used for preceding generalised pruritus. The majority of patients were treated with topical agents including calcineurin inhibitors and steroids with variable response. Of the 41 cases, 25 (61.0%) had no response with 14 (34.1%) showing some improvement. One case had total resolution with photoprotection measures, tacrolimus ointment, and cessation of CDK 4/6 inhibitor treatment [15]. Five patients additionally received oral corticosteroids of which two reported some improvement [6]. Three patients also had UVB phototherapy. Ruxolitinib cream has also been suggested as a potential treatment option in a case series of 10 patients after it showed some repigmentation effect [16]. Cessation of CDK 4/6 inhibitor led to improvement in lesions in just two of five reported patients. More clinical experience and studies are required to evaluate the role of topical and oral options that have so far shown poor outcomes. Of 19 reported cases, 14 patients persisted with treatment with cessation in only five, suggesting that most physicians choose to discuss with their patients to treat this toxicity, weighing the associated risks and benefits.

We suggest the following management principles. Physicians can stem the progress of VLD by targeting the ongoing inflammatory process, by means of topical agents such as corticosteroids, Janus kinase (JAK) inhibitors, and calcineurin inhibitors, before considering oral immunosuppressants like oral corticosteroids. Reducing further damage by way of sun avoidance should be advised. VLD areas might also be more susceptible to sunburn since depigmented areas no longer have the same melanin protection as normal skin. One also has to weigh the risks and benefits of CDK4/6 inhibitors for the patient’s underlying malignancy and discuss alternative agents versus treating through.

Narrowband UVB therapy locally immunosuppresses and stimulates the remaining melanocytes to produce more melanin and is used in classic vitiligo. However, given the proposed pathophysiology of UVB radiation compounding DNA damage, it remains to be seen if it is truly helpful for VLD and should be used with caution, especially in patients with lesions over photoexposed sites.

The clinical and disease characteristics, treatment course, and nature of VLD of the 41 patients included in our literature review have been summarised in Table 1. 

**Table 1:** Patient and disease characteristics, treatment, and outcome CDK: cyclin-dependent kinase, VLD: vitiligo-like depigmentation, NR: no response, PR: partial response

Patient number/ age	CDK4/6 inhibitor and hormone partner	Progression-free survival (months)	Sites of VLD	Months on treatment before onset	Treatment for VLD	Outcome of dermatological treatment
1/52 [17]	Palbociclib/not specified	~	Abdomen, legs	7	Topical corticosteroids and calcineurin inhibitor	NR
2/78 [9]	Ribociclib/not specified	~	Face, abdomen, chest, arms, legs, hands and feet	7	Oral steroids	PR
3/61 [16]	Ribociclib/letrozole	>14	Face, trunk, arms	1	Topical corticosteroids and tacrolimus ointment	NR
4/51 [16]	Ribociclib/letrozole	>10	Trunk, arms	1	Topical corticosteroids	NR
5/86 [16]	Abemaciclib/anastrozole	15	Trunk	14	Nil	NR
6/69 [16]	Palbociclib/letrozole	4	Legs	18	Nil	NR
7/58 [16]	Palbociclib/letrozole	48	Arms, legs	6	Nil	NR
8/50 [16]	Ribociclib/tamoxifen	31	Face, arms	13	Tacrolimus ointment, ultraviolet-B (UVB) phototherapy	NR
9/73 [16]	Ribociclib/exemestane	24	Face, trunk, arms, legs	28	Topical corticosteroids	NR
10/42 [16]	Abemaciclib/anastrozole	>52	Arms	24	Topical calcineurin inhibitors	NR
11/49 [16]	Abemaciclib/letrozole	>29	Face	5	Nil	NR
12/37 [16]	Palbociclib/fulvestrant	>39	Face	1	Desonide ointment UVB therapy, tacrolimus ointment, ruxolitinib cream	NR
13/71 [13]	Ribociclib/letrozole	15	Face, back, arms, legs	7	~	NR
14/54 [13]	Ribociclib/letrozole	31	Face, trunk, arms	3	~	PR
15/58 [18]	Palbociclib/letrozole	>24	Back, axillary fossa, arms, legs, hands and feet	10	Nil	NR
16/51 [12]	Ribociclib/letrozole	~	Face, neck, arm	10	Topical corticosteroids and calcineurin inhibitors	PR
17/50 [8]	Ribociclib/anastrozole	~	Face, arms	18	Topical corticosteroids and tacrolimus ointment	~
18/55 [14]	Ribociclib then palbociclib/letrozole	>52	Trunk, arms, legs	3	Oral and topical corticosteroids	NR
19/80 [14]	Ribociclib/letrozole	39	~	12	~	NR
20/41 [15]	Palbociclib/fulvestrant	~	Neck, arms, hands	2	Tacrolimus ointment	Total resolution
21/47 [19]	Palbociclib/letrozole	~	Chest, arms, legs	1	Nil	NR
22/71 [3]	Ribociclib/letrozole	~	Face, arms, feet	5	Topical corticosteroids	PR
23/66 [6]	Ribociclib/letrozole	~	Chest, arms	5	Topical corticosteroids	PR
24/67 [6]	Ribociclib/letrozole	~	Neck, chest, arms	4	Topical corticosteroids	NR
25/62 [6]	Ribociclib/letrozole	~	Trunk, arms	10	Topical corticosteroids and calcinuerin inhibitors	PR
26/73 [6]	Ribociclib/letrozole	~	Face, chest, back, arms, legs	5	Topical corticosteroids and UVB phototherapy	NR
27/53 [6]	Ribociclib/letrozole	~	Chest. back. arms	9	Topical corticosteroids	PR
28/71 [6]	Ribociclib/letrozole	~	Back, arms	12	Nil	NR
29/79 [6]	Ribociclib/letrozole	~	Face, arms, hands	16	Oral and topical corticosteroids	PR
30/66 [6]	Ribociclib/letrozole	~	Face, arms. hands and feet	6	Topical corticosteroids	PR
31/54 [6]	Ribociclib/letrozole	~	Face, chest, back, arms, hands and feet	8	Oral and topical corticosteroids	PR
32/48 [6]	Ribociclib/letrozole	~	Chest, arms	4	Topical corticosteroids	PR
33/73 [6]	Ribociclib/letrozole	~	Chest, back, hands	16	Topical corticosteroids	PR
34/59 [6]	Ribociclib/letrozole	~	Arms, hands	12	Topical corticosteroids	NR
35/57 [6]	Ribociclib/letrozole	~	Trunk, arms, legs	10	Calcineurin inhibitors	PR
36/40 [6]	Ribociclib/letrozole	~	Face, chest, back	9	Calcineurin inhibitors and oral steroids	NR
37/42 [6]	Palbociclib/fulvestrant	~	Face, arms, legs and hands	8	Calcineurin inhibitors	PR
38/63 [6]	Palbociclib/letrozole	~	Face, chest, back, arms	12	Topical corticosteroids	NR
39/70 [20]	Ribociclib/letrozole	~	Face, neck	8	~	NR
40/56 [4]	Ribociclib/anastrozole	~	Neck, chest, back, arms, hands	6	Topical immunosuppressants and oral steroids	NR
41/72 (current case)	Ribociclib then palbociclib/fulvestrant	>19	Chest, back, legs	11	Topical corticosteroids and tacrolimus ointment	NR

The full list of resources used in our literature review [3,4,6,8-9,12-20] can be found in Table 2.

**Table 2:** List of resources included in the literature review CDK: cyclin-dependent kinase

Author	Year	Title	Number of cases
Algethami et al. [17]	2024	Palbociclib-induced vitiligo-like lesions: a report of a challenging case	1
Anjaneyan et al. [9]	2022	Ribociclib-induced extensive vitiligo-like lesions: possible pathomechanisms with clinical, dermoscopic and histological correlation	1
Bang et al. [16]	2024	Multi‑centre retrospective review of vitiligo‑like lesions in breast cancer patients treated with cyclin‑dependent kinase 4 and 6 inhibitors	10
Chan et al. [13]	2022	Drug-induced vitiligo-like depigmentation from a CDK 4/6 inhibitor	2
Gao et al. [18]	2023	Vitiligo-like lesions induced by cyclin-dependent kinase 4/6 inhibitor palbociclib: a case report and literature review	1
Kothari et al. [12]	2025	Ribociclib-induced vitiligo-like lesions	1
Menteşoğlu [8]	2023	Photoallergic dermatitis and vitiligo-like lesion in a patient with metastatic breast cancer using ribociclib	1
Pasqualoni et al. [14]	2023	Case report: vitiligo-like toxicity due to ribociclib during first-line treatment of metastatic breast cancer: two cases of premature interruption of therapy and exceptional response	2
Perez et al. [15]	2024	Vitiligo-like depigmentation affecting sun-exposed areas caused by palbociclib	1
Romagnuolo et al. [19]	2023	Vitiligo-like lesions induced by cyclin-dependent kinase 4/6 inhibitor palbociclib	1
Sharaf et al. [3]	2022	Vitiligo-like lesions in a patient with metastatic breast cancer treated with cyclin-dependent kinase (CDK) 4/6 inhibitor: a case report and literature review	1
Sollena et al. [6]	2021	Vitiligo‑like lesions in patients with advanced breast cancer treated with cyclin‑dependent kinases 4 and 6 inhibitors	16
Silvestre Torner et al. [20]	2022	Ribociclib-induced vitiligo: a case report	1
Turkel et al. [4]	2023	Vitiligo-like lesions associated with ribociclib in a woman with metastatic breast cancer	1

## Conclusions

Given the expanding role of CDK 4/6 inhibitors that now extend beyond just the metastatic setting, physicians are more likely to gain exposure to the rarer side effects of these drugs. Vitiligo-like lesions whilst not typically a threat to mortality, negatively impact compliance, tolerability and patient’s quality of life. There is a paucity of information regarding diagnosis, treatment, and need for discontinuation, or if dose adjustment and switch of CDK 4/6 inhibitors are potential mitigating strategies. Our article sheds some light on identifiable diagnostic hallmarks and possible therapeutic options available. Continuation of the drug might be possible after discussion with the patient and their willingness to accept VLD as an adverse effect. Physicians should counsel patients about this potential toxicity, observe for it and preceding pruritus, and consider an early referral to dermatology should it develop. More research is necessary to improve the understanding and management of this condition, as well as to enhance patient care and oncological outcomes.
